# Clinical attitudes and perceived barriers to early mobilization of critically ill patients in adult intensive care units

**DOI:** 10.5935/0103-507X.20180037

**Published:** 2018

**Authors:** Paula Caitano Fontela, Luiz Alberto Forgiarini Jr., Gilberto Friedman

**Affiliations:** 1 Programa de Pós-Graduação em Ciências Pneumológicas, Faculdade de Medicina, Universidade Federal do Rio Grande do Sul - Porto Alegre (RS), Brasil.; 2 Programa de Pós-Graduação em Biociências e Reabilitação e Reabilitação e Inclusão, Centro Universitário Metodista IPA - Porto Alegre (RS), Brasil.

**Keywords:** Early ambulation, Respiration, artificial, Muscle weakness, Patient care team, Physical therapy modalities

## Abstract

**Objective:**

To investigate the knowledge of multi-professional staff members about the early mobilization of critically ill adult patients and identify attitudes and perceived barriers to its application.

**Methods:**

A cross-sectional study was conducted during the second semester of 2016 with physicians, nursing professionals and physical therapists from six intensive care units at two teaching hospitals. Questions were answered on a 5-point Likert scale and analyzed as proportions of professionals who agreed or disagreed with statements. The chi-square and Fisher's exact tests were used to investigate differences in the responses according to educational/training level, previous experience with early mobilization and years of experience in intensive care units.

**Results:**

The questionnaire was answered by 98 out of 514 professionals (response rate: 19%). The acknowledged benefits of early mobilization were maintenance of muscle strength (53%) and shortened length of mechanical ventilation (83%). Favorable attitudes toward early mobilization included recognition that its benefits for patients under mechanical ventilation exceed the risks for both patients and staff, that early mobilization should be routinely performed via nursing and physical therapy protocols, and readiness to change the parameters of mechanical ventilation and reduce sedation to facilitate the early mobilization of patients. The main barriers mentioned were the unavailability of professionals and time to mobilize patients, excessive sedation, *delirium*, risk of musculoskeletal self-injury and excessive stress at work.

**Conclusion:**

The participants were aware of the benefits of early mobilization and manifested attitudes favorable to its application. However, the actual performance of early mobilization was perceived as a challenge, mainly due to the lack of professionals and time, excessive sedation, *delirium*, risk of musculoskeletal self-injury and excessive stress at work.

## INTRODUCTION

A growing body of evidence supports the safety, feasibility and long-term functional benefits of early physical therapy, i.e., starting within the first 48 hours of mechanical ventilation (MV) and being maintained throughout the stay in the intensive care unit (ICU).^(^^1^^-^^8^^)^ Its potential benefits notwithstanding, effective early mobilization (EM) is not widely performed in the ICU. International multicenter studies on EM in the ICU evidence a low prevalence of out-of-bed mobilization, especially among patients under MV.^(^^9^^,^^10^^)^ The same situation was recently described in Brazilian ICUs, where only 10% of patients under MV were mobilized out of bed.^(^^11^^)^

Few studies have sought to explain why EM is not effectively performed in ICU clinical practice. Some studies on improvement of the quality of care delivery investigated whether the attitudes and education of professionals relative to EM are barriers to actual performance.^(^^12^^-^^14^^)^ These studies identified personal and patient safety and lack of clinical comprehension as potentially relevant hindrances to the performance of EM. Recent studies^(^^15^^-^^17^^)^ found that the need of a larger number of professionals, insufficient working hours and the staff's culture regarding mobilization, including a lack of resources, prioritization and leadership, are among the main interdisciplinary barriers to the performance of EM.

A multicenter prevalence study found that the EM of patients under MV is uncommon, especially in regard to patients ventilated through endotracheal tubes, with muscle weakness, cardiovascular instability and sedation being the most commonly perceived barriers to mobilizing patients at a higher level. These difficulties might be overcome, which is relevant to increasing mobilization in Brazilian ICU.^(^^11^^)^

The aim of the present study was to investigate the knowledge of a multi-professional team on the EM of critically ill adult patients and identify their attitudes and perceived barriers to effective performance.

## METHODS

The present cross-sectional study consisted of a survey of professionals who deliver care at six ICUs in two teaching hospitals in Brazil. The study was conducted in the second semester of 2016 and was approved by the research ethics committees of the participating hospitals, *Hospital de Clínicas de Porto Alegre* (HCPA; 1.335.156) and *Irmandade da Santa Casa de Misericórdia de Porto Alegre* (ISCMPA; 1.647.299). Informed consent was obtained through electronic means before the electronic questionnaire was answered.

All the professionals at the ICU of both hospitals were invited to participate in the study through e-mails sent by the study coordinator to service chairs, who then resent them to the professionals. Physicians, including routine and assisting physicians and medical residents, were named by the medical team chair of each ICU. Nurses, nursing technicians and physical therapists allocated to these units were named by the nursing team chair of each service and the chair of the department of physical therapy of each hospital.

The link to access the questionnaire was sent by e-mail to the service chairs together with the invitation to participate in the study. The service chairs resent the e-mails to the members of their teams, on which the study coordinator was copied. To make responding to the questionnaire and data collection easier, it was developed using the software SurveyMonkey^®^, and the results were obtained in real-time through coupling to Statistical Package for the Social Sciences (SPSS) software.

The questionnaire was adapted from the one employed in a recent study,^(^^15^^)^ which was applied to the full intensive care team. The questionnaire included items to investigate the respondents' knowledge about the potential benefits of EM in the ICU, their attitudes regarding the application of this technique in the ICU and perceived barriers to the performance of EM. The items were answered on a 5-point Likert scale with the following options: "I fully agree", "I agree", "I neither agree nor disagree", "I disagree" and "I fully disagree".

Early mobilization was defined as any activity performed beyond the range of motion within 48 hours of the onset of MV. Experience with EM and availability of an EM protocol in the ICU were defined as present when the respective responses to the following questions were "yes": (1) "Have you had training in, have you worked at or do you work at an institution where patients under MV are actively mobilized?"; and (2) "Has an EM protocol been implemented at the ICU where you work?"

The right answers to the questions investigating knowledge about EM were defined before the onset of the survey. The answers "I disagree" and "I fully disagree" were considered the right ones for the question "Does range of motion suffice to maintain muscle strength in the ICU?" The answers "I agree" and "I fully agree" were considered the right ones for the item on whether EM is associated with a shorter duration of MV. For the remainder of the items, positive responses were "I agree" or "I fully agree" and negative answers were "I neither agree nor disagree", "I disagree" or "I fully disagree".

The questionnaire for physicians included a non-hierarchical list of potential barriers to mobilization in the ICU, including the option "other (specify)", as follows: (1) duration of nursing procedures, (2) duration of respiratory physical therapy, (3) availability of physical therapists, (4) patient undergoing procedures, (5) excessive sedation, (6) mobility is irrelevant in the ICU, (7) *delirium*, (8) access to specialized equipment, (9) personal safety, (10) patient safety, (11) cost and (12) therapy is not performed although it is recommended. The questionnaires for nurses and physical therapists also included a list, with the following items: (1) risk of musculoskeletal self-injury, (2) fatigue, (3) excessive stress at work, (4) need to work overtime, (5) other (specify). In both questionnaires, the professionals could mark any number of options they considered appropriate and add other items they held to represent potential hindrances to EM in the ICU.

The participants were given 1 month to respond to the questionnaire from the moment it was sent. An e-mail reminding the participants to respond to the questionnaire was sent one week before the deadline. To ensure that no participant would be included in the survey twice, e-mail addresses were checked against the list of participants' e-mail addresses. The questionnaires were answered anonymously and on a voluntary basis.

Descriptive statistics were performed to characterize the sample. The responses given on the Likert scale ware expressed as absolute frequencies and proportions. The chi-square test was used to investigate whether the physicians' responses differed as a function of their educational level (medical residency versus master's degree versus doctoral degree). Fisher's exact test was used to investigate significant differences in the nursing staff's responses as a function of their educational level (nursing technicians versus nurses), previous experience with EM for physicians, nursing professionals and physical therapists (yes versus no), and years of experience (< 5 years versus ≥ 5 years) for nursing professionals and physical therapists. The significance level was set at p < 0.05. The data were stored and analyzed using SPSS software for Windows, version 18.0.

## RESULTS

Both participating institutions were university-affiliated hospitals, and the ICU types were as follows: clinical-surgical (n = 3), pneumological (n = 1), oncological (n = 1) and transplant (n = 1). A total of 514 professionals were invited to participate, including 154 physicians, 293 nursing professionals and 67 physical therapists.

### Results relative to the questionnaire for physicians

Twenty-two physicians responded to the questionnaire, corresponding to a response rate of 14% (22/154). All the physicians were intensivists, and medical residency was the most prevalent educational level (Table 1). Most physicians reported having had previous experience with EM and responded that range of motion is insufficient to preserve the muscle strength of critically ill patients (n = 12; 55%) and that EM shortens the length of MV (n = 19; 86%) (Table 2), without any significant differences according to educational level or previous experience with EM.

**Table 1:** Professionals’ characteristics and experience with early mobilization

	n (%)
Physicians	n = 22
Medical residency	11 (50)
Master’s degree	5 (23)
Doctoral degree	6 (27)
Experience with EM	19 (86)
Nursing team*	n = 61
< 5 years of experience in the ICU	8 (13)
≥ 5 years of experience in the ICU	53 (87)
Experience with EM	34 (56)
Physical therapists	n = 15
< 5 years of experience in the ICU	4 (27)
≥ 5 years of experience in the ICU	11 (73)
Experience with EM	11 (73)

EM - early mobilization; ICU - intensive care unit.

* 32 (53%) nurses and 29 (47%) nursing technicians.

**Table 2:** Knowledge about the potential benefits of early mobilization in the adult intensive care unit per professional category and educational/training level

	Disagreed n (%)
ROM suffices to preserve muscle strength in the ICU	52 (53)
Physicians	p = 0.284
Medical residency (n = 11)	6 (55)
Master’s degree (n = 5)	4 (80)
Doctoral degree (n = 6)	2 (33)
Nursing team*	p = 0.255
< 5 years of experience in the ICU (n = 8)	2 (25)
≥ 5 years of experience in the ICU (n = 53)	28 (53)
Physical therapists	p = 0.560
< 5 years of experience in the ICU (n = 4)	2 (50)
≥ 5 years of experience in the ICU (n = 11)	8 (73)
	**Agreed** **n (%)**
Early mobilization shortens the length of MV	81 (83)
Physicians	p = 0.099
Medical residency (n = 11)	8 (73)
Master’s degree (n = 5)	5 (100)
Doctoral degree (n = 6)	6 (100)
Nursing team*	p = 0.762
< 5 years of experience in the ICU (n = 8)	7 (88)
≥ 5 years of experience in the ICU (n = 53)	40 (75)
Physical therapists	**
< 5 years of experience in the ICU (n = 4)	4 (100)
≥ 5 years of experience in the ICU (n = 11)	11 (100)

ROM - range of motion; ICU - intensive care unit; MV - mechanical ventilation.

* 32 (53%) nurses and 29 (47%) nursing technicians. p-value calculated by means of the chi-square test to compare educational level between agreement and disagreement among physicians, and by means of the Fisher’s exact test to compare years of experience in the intensive care unit between agreement and disagreement among nurses and physical therapists.

** p-value was not calculated because the variable is a constant.

**Figure 1:**
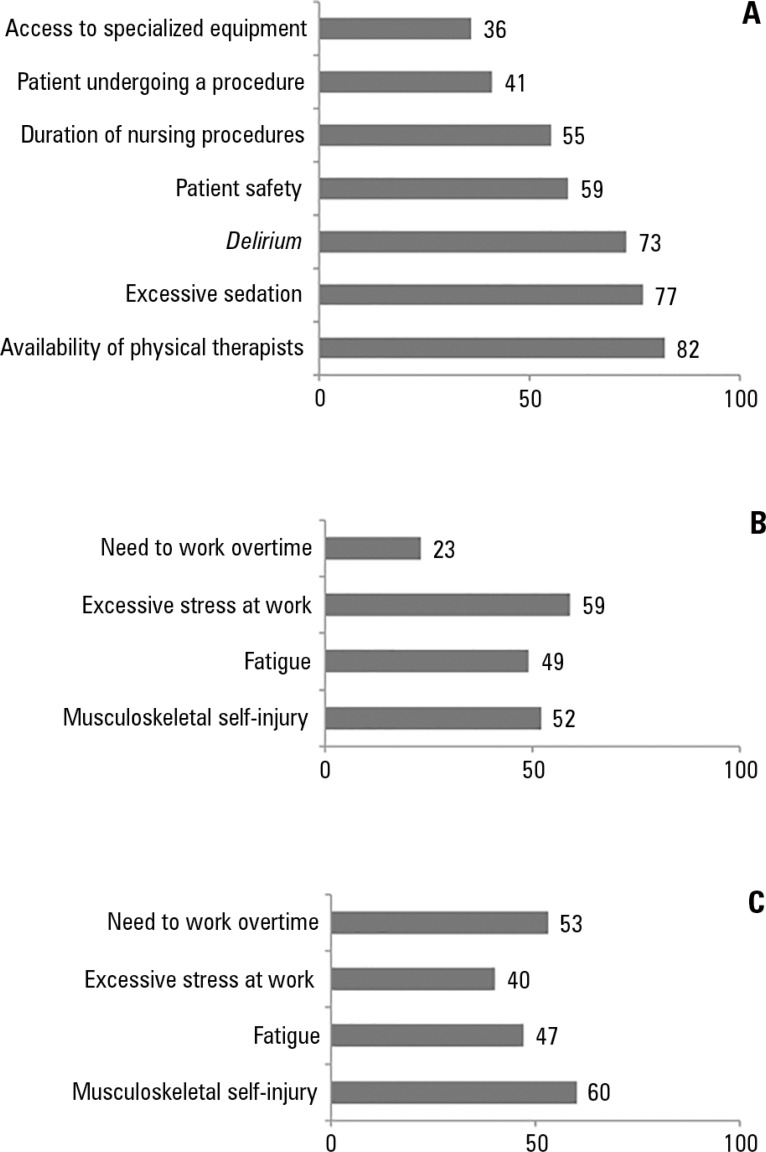
Barriers reported by the professionals (A - physicians; B - nurses and nursing technicians; C - physical therapists) to early mobilization of critically ill adult patients.

Twenty-one (95%) physicians agreed that the benefits of EM exceed the risks for patients under MV (Table 3). Most physicians stated they would allow EM for patients under MV (n = 20; 91%) and that they would agree to change the MV parameters (n = 19; 86%) and reduce the level of sedation to enable EM (n = 21; 95). Ten (45%) physicians did not agree with EM for patients receiving vasoactive drugs. Eighteen out of 22 physicians who responded to the questionnaire stated that EM should be routinely performed via nursing and physical therapy protocols unless explicitly contraindicated. The responses did not significantly differ in regard to educational level or previous experience with EM. The barriers to EM most frequently indicated by the physicians are described in figure 1A.

**Table 3:** Physicians’ attitudes relative to the indication of early mobilization in the adult intensive care unit per educational level

Instrument item	Agreed n (%)
The benefits of EM exceed the risks for patients under MV	p = 0.488
Medical residency (n = 11)	10 (91)
Master’s degree (n = 5)	5 (100)
Doctoral degree (n = 6)	6 (100)
I would agree with the EM of patients receiving vasopressors	p = 0.674
Medical residency (n = 11)	5 (45)
Master’s degree (n = 5)	3 (60)
Doctoral degree (n = 6)	4 (67)
I would agree with the EM of patients under MV	p = 0.428
Medical residency (n = 11)	10 (91)
Master’s degree (n = 5)	4 (80)
Doctoral degree (n = 6)	6 (100)

EM - early mobilization; MV - mechanical ventilation. p-value calculated by means of the chi-square test to compare educational level between agreement and disagreement.

### Results relative to the questionnaire for the nursing staff

Sixty-one members of the nursing team responded to the questionnaire, corresponding to a response rate of 21% (61/293). Of these, 29 (47%) were nursing technicians. Most nursing professionals reported having more than 5 years of experience in the ICU, and most nurses had a specialization in intensive care (n = 33; 43%). Twenty-seven (44%) respondents reported no previous experience with EM in the ICU (Table 1). Half of this group stated that range of motion is insufficient to preserve the muscle strength of critically ill patients (n = 30; 49%), and most stated that EM shortens the length of MV (n = 47; 77%) (Table 2). The responses did not significantly differ according to years of experience in the ICU, educational level or previous experience with EM.

Most nursing professionals agreed that the benefits of EM exceed the risks for patients under MV (n = 42; 69%). Nursing staff with more than 5 years of experience in the ICU were more likely to agree that the benefits of EM exceed the risks for patients under MV (p = 0.049) (Table 4). Most respondents stated that they had enough time to help mobilize patients under MV (n = 38; 62%) and that the benefits of EM for patients under MV exceed the risks to the team's personal and professional safety (n = 43; 70%). The nursing technicians were less likely to agree that they had enough time to help mobilize patients under MV compared with the nurses (n = 14; 48% and n = 24; 75%, respectively; p = 0.038). The responses did not differ regarding the respondents' previous experience with EM.

**Table 4:** Nursing professionals’ and physical therapists’ attitudes relative to the indication of early mobilization in the adult intensive care unit per educational/training level

Instrument item	Agreed n (%)
The benefits of EM exceed the risks for patients under MV	56 (74)
Nursing team*	p = 0.049
< 5 years of experience in the ICU (n = 8)	8 (100)
≥ 5 years of experience in the ICU (n = 53)	34 (64)
Physical therapists	p = 1.0
< 5 years of experience in the ICU (n = 4)	4 (100)
≥ 5 years of experience in the ICU (n = 11)	10 (91)
I agree that I have enough time to help mobilize a patient under MV once per day	48 (63)
Nursing team*	p = 0.698
< 5 years of experience in the ICU (n = 8)	6 (75)
≥ 5 years of experience in the ICU (n = 53)	32 (60)
Physical therapists	p = 0.077
< 5 years of experience in the ICU (n = 4)	1 (25)
≥ 5 years of experience in the ICU (n = 11)	9 (82)
I agree that the benefits of EM for patients under MV exceed the risks for the staff	56 (74)
Nursing team*	p = 0.091
< 5 years of experience in the ICU (n = 8)	8 (100)
≥ 5 years of experience in the ICU (n = 53)	35 (66)
Physical therapists	p = 0.476
< 5 years of experience in the ICU (n = 4)	3 (75)
≥ 5 years of experience in the ICU (n = 11)	10 (91)

EM - early mobilization; MV - mechanical ventilation; ICU - intensive care unit.

* 32 (53%) nurses and 29 (47%) nursing technicians. p-value calculated by means of the Fisher’s exact test to compare years of experience in the intensive care unit between agreement and disagreement among nurses and physical therapists.

The barriers to EM most frequently indicated by the nursing professionals are described in figure 1B.

### Results relative to the questionnaire for the physical therapists

Fifteen physical therapists responded to the questionnaire, corresponding to a response rate of 22% (15/67). Most respondents (73%) reported having more than 5 years of experience in the ICU and previous experience with EM (Table 1), being that the largest proportion had a specialization in intensive care (n = 7; 47%). Most physical therapists stated that range of motion is insufficient to preserve the muscle strength of critically ill patients in the ICU (n = 10; 67%), and all agreed that EM shortens the length of MV (Table 2), without differences according to years of experience in the ICU or previous experience with EM.

Almost all the physical therapists agreed that the benefits of EM exceed the risks for patients under MV (n = 14; 93%), and that the benefits of EM for patients under MV exceed the risks to the team's personal and professional safety (n = 13; 87%). Most respondents (n = 10; 67%) stated that they had enough time to help mobilize patients under MV (Table 4). The responses did not differ regarding years of experience in the ICU. The physical therapists with previous experience with EM were more likely to agree that the benefits of EM for patients under MV exceed the risks to the team's personal and professional safety (p = 0.050).

The barriers to EM most frequently indicated by the physical therapists are described in figure 1C.

## DISCUSSION

Among the main findings of the present study conducted in the ICU of two Brazilian teaching hospitals, we highlight that most members of the multi-professional team had knowledge about the potential benefits of EM, including the maintenance of muscle strength and a shorter duration of MV, and that most participants agreed that the benefits of EM exceed the risks to patients under MV. Similar results were reported in a previous study^(^^15^^)^ that analyzed the knowledge and attitudes of multi-professional health team members involved in care delivery to critically ill patients.

Most physicians agreed on the EM of patients under MV; however, only half of them agreed on indicating EM for patients receiving vasoactive drugs. The physicians stated they would change the MV parameters and reduce sedation to enable the EM of patients.^(^^15^^)^ Approximately two-thirds of the physical therapists and nursing professionals stated they had sufficient time to help mobilize patients under MV once per day. Most physical therapists and nursing professionals agreed that the benefits of EM for patients under MV exceed the risks to the team's personal and professional safety. Nursing technicians were less likely to agree that they had sufficient time to help mobilize patients under MV once per day compared to nurses. The barriers to EM most frequently cited by physicians were the unavailability of professionals on the team and of sufficient time to routinely mobilize patients, excessive sedation and *delirium.*^(^^15^^,^^17^^)^ Risk of musculoskeletal self-injury and excessive stress at work were also mentioned by nurses and physical therapists as barriers to EM.

The findings of the present study confirm the hypothesis that there is a gap between evidence-based knowledge and its application in clinical practice. Several authors admit that while knowledge continues to advance, practice remains one step behind.^(^^18^^,^^19^^)^ The multi-professional participants in the present study exhibited knowledge about the potential benefits of and a favorable attitude toward EM in the ICU but identified several barriers to its actual application in clinical practice. The barriers to EM are patient-related, such as patient symptoms and conditions; structural, such as human and technical resources; related to the ICU culture, including habits and the particular attitudes at each institution; and process-related, from lack of coordination to lack of rules for the distribution of tasks and responsibilities.^(^^20^^)^ These multiple barriers were also detected in the present study.

More than 80% of the physicians stated that EM should be routinely performed via nursing and physical therapy protocols, unless explicitly contraindicated. In addition, they stated they would agree to change MV parameters and reduce sedation to enable the EM of patients. Nurse-oriented mobility protocols point to increased mobility and functional benefits for patients.^(^^21^^,^^22^^)^ However, the workload of the ICU nursing team is admittedly high, which might impact safety and the quality of care delivered.^(^^23^^,^^24^^)^ These facts confirm the results of the present study, as only 62% of the nurses agreed that they had sufficient time to help mobilize patients under MV once per day.

Although most nursing professionals and physical therapists agreed that they had sufficient time to help mobilize patients under MV once per day, the need to work overtime was one of the main barriers to EM that they mentioned. The unavailability of physical therapists was the main barrier to EM mentioned by the participating physicians. These findings confirm the ICU culture- and process-related barriers already established in the literature.^(^^20^^)^

Several barriers were mentioned by all the groups of participants, including the unavailability of professionals and insufficient time to perform EM with critically ill patients. These barriers were also reported by members of multi-professional teams in the United States^(^^15^^)^ and Canada.^(^^17^^)^ Time and the professionals required to mobilize critically ill patients might be considerable hindrances to EM in the ICU. In addition, they represent a frequently reported concern in regard to the improvement of the quality of care needed to facilitate the acceptance of mobilization.^(^^12^^-^^15^^)^ A solution developed at some centers was to shift the perception and revise priorities in the daily care delivery routine to include mobilization.^(^^1^^,^^25^^,^^26^^)^ Creation and implementation of a dedicated ICU mobility team might also represent an option to increase the mobility of patients and was proven safe and viable. This approach allowed the mobilized patients to get out of bed on 2.5 more days, without any adverse events, resulting in better clinical outcomes and functional independence, in addition to reducing hospital costs.^(^^27^^)^

Concerns about musculoskeletal self-injury, stress and overtime work were barriers mentioned by the nursing professionals and physical therapists who participated in the present study; these findings corroborate the reports in the literature.^(^^15^^)^ Although EM was shown to be safe and feasible for patients, there is no information in regard to the staff safety, which might constitute a considerable barrier to EM in the ICU.^(^^28^^)^

Our study has potential limitations. First, the results are subjected to selection bias as a function of the low response rate. Second, the fact that we did not calculate the sample size needed to ensure that the number of participants was sufficient to detect significant differences might have resulted in a type II error in the data analysis. Finally, the responses to the questions investigating "knowledge" might have been influenced by the fact that the literature on EM is scarce and reduced the potential for the generalization of clinical trials on EM. As strengths, the present was the first study that investigated the full staff that provides care to critically ill patients at academic institutions, including nursing technicians, to better understand interdisciplinary concerns about EM.

## CONCLUSION

Most participants had information about the benefits and significance of early mobilization for critically ill patients and exhibited a favorable attitude toward the performance of early mobilization in the intensive care unit. However, they mentioned countless barriers related to the work routine, staff interaction, unit operation and clinical conditions of patients. Early mobilization in the intensive care unit was perceived as a challenge, mainly due to the lack of professionals, insufficient time, excessive sedation, *delirium*, risk of musculoskeletal self-injury and excessive stress at work. We detected considerable barriers to the early mobilization of critically ill adult patients admitted to the intensive care unit. This information might serve to initiate the training of professionals involved in this procedure and in the implementation of institutional protocols.
